# How does physical education influence university students’ psychological health? An analysis from the dual perspectives of social support and exercise behavior

**DOI:** 10.3389/fpsyg.2025.1457165

**Published:** 2025-02-18

**Authors:** Xu Han, Haozhen Li, Ling Niu

**Affiliations:** ^1^Department of Sports, Beijing Institute of Graphic Communication, Beijing, China; ^2^Department of Physical Education, Zhengzhou University (Main Campus), Zhengzhou, China

**Keywords:** physical education, mental health, social support, exercise behavior, chain

## Abstract

**Objective:**

Physical education, as a central component of educational systems, plays a unique role in enhancing the psychological well-being of university students. This study investigates the impacts of physical education on students’ mental health and examines the mediating roles of social support and exercise behavior.

**Methodology:**

A cross-sectional survey was conducted using the Physical Education Satisfaction Scale, SCL-90, the Social Support Questionnaire, and the International Physical Activity Questionnaire. A total of 1,437 university students were assessed.

**Results:**

The research found a positive correlation between physical education at universities and students’ mental health, with a direct significant effect (*β* = 0.622, *p* < 0.001). Moreover, physical education positively influenced social support and exercise behavior (*β* = 0.523, *p* < 0.001; *β* = 0.473, *p* < 0.001). In turn, social support significantly fostered exercise behavior and improved mental health (*β* = 0.578, *p* < 0.001; *β* = 0.277, *p* < 0.001). Additionally, enhanced exercise behavior positively contributed to better mental health (*β* = 0.357, *p* < 0.001).

**Conclusion:**

Physical education is a crucial influencing factor for university students’ psychological health and also indirectly impacts mental well-being through improved social support and increased exercise behavior.

## 1 Introduction

Mental health refers not only to the absence of psychological disorders but also to positive mental behaviors, such as maintaining physical well-being and engaging in healthy social interactions. According to data from the National Bureau of Statistics of China, the total enrollment in general undergraduate and vocational diploma programs reached 10.42 million, representing a 2.73% increase from the previous year (Meng, 2024). This large population faces growing pressures due to the fast-paced societal environment and the challenging job market. Surveys indicate that over one-fifth of Chinese college students experience some degree of psychological distress, with the prevalence of mental disorders ranging from 10 to 30% (Liu et al., 2023). Amid the current prominence of mental health issues, enhancing the mental well-being and resilience of college students is of paramount importance. With the advancement of national initiatives such as “Healthy China” and “National Fitness,” physical education is increasingly recognized as a vital means of improving students’ mental health.

Physical education, as an essential component of student life, plays a vital role not only in improving physical fitness but also in promoting psychological health (Zhu and Li, 2022). Through participation in physical activities, students can enhance their physical strength and overall health while positively influencing their mental well-being by alleviating stress, boosting self-confidence, and increasing self-efficacy (Gkintoni et al., 2024). Moreover, engaging in sports provides students with opportunities to interact and collaborate with others. The social interactions and teamwork involved help develop interpersonal skills and foster a strong network of social support (Opstoel et al., 2020). Significant research, such as the study by Ma et al. (2021), which surveyed 5,265 university students, has revealed a considerable correlation between physical literacy, mental health, and resilience. Zhang and Min (2022) found that different physical exercises significantly affect the physical and mental health of female university students, though their impact on social support was not significant.

Social support, as an important psychological resource, has a significant impact on stress management and mental health maintenance (Kleemann et al., 2020; Wang et al., 2022). Physical education provides opportunities for teamwork and social interaction, helping students build extensive social networks (Yang et al., 2024). In participating in group sports activities, students have the chance to meet new friends and strengthen connections with their peers, thereby gaining crucial emotional support and friendships that help reduce feelings of loneliness and social isolation. Through teamwork in sports activities, students can experience a sense of belonging and group identity, which is essential for maintaining mental health (Huang et al., 2022; Zheng et al., 2024). Moreover, exercise behavior has been affirmed by numerous studies as an effective mediator for mental health. Regular physical activity enhances psychological resilience, emotional regulation, and overall well-being (Fox, 1999). Although existing literature has focused on the direct link between physical exercise and mental health, only a few studies have considered the influence of social support on this relationship. Studies employing a chain mediation model—whereby physical education impacts psychological health through social support and subsequently exercise behavior—are relatively scarce. This model comprehensively reflects how physical education can improve mental health through multi-level influences.

Against this backdrop, this paper aims to explore the relationship between physical education and student mental health, particularly focusing on the chained mediating roles of social support and exercise behavior. Employing survey and empirical analysis methods with university students as subjects, this study analyzes how physical education can enhance social support and stimulate exercise behavior, thereby influencing their psychological health levels. Grounded in social cognitive theory and behavioral development theory, this research attempts to substantiate the effectiveness of physical education in promoting student mental health, thereby providing theoretical foundations and practical guidance for the formulation and implementation of physical education policies.

## 2 Mechanisms and hypothesis

### 2.1 The relationship between physical education and student mental health

In contemporary society, the role of physical education should extend beyond merely enhancing students’ athletic skills and physical fitness; it plays a crucial role in fostering and maintaining mental health (Zhu and Li, 2022). Engaging in physical activities provides students with an outlet to relieve academic and interpersonal pressures and simultaneously bolsters social skills and a sense of collective honor through experiences in sports competitions and teamwork, thereby exerting a positive psychological impact.

Physical activities influence mental health through multiple mechanisms. Firstly, regular exercise effectively promotes cerebral blood circulation, which improves brain oxygen and nutrient supply, alleviating mental fatigue and enhancing cognitive clarity (Jia et al., 2022). Secondly, physical activity triggers the production of endorphins and other natural painkillers and mood enhancers, which elevate mood, reduce anxiety and depression, and add enjoyment to life (Dinas et al., 2011). Furthermore, team sports serve as an excellent social platform, providing an atmosphere of common goals and collaboration that helps students enhance interpersonal relationships and group communication skills while realizing personal values (Grehaigne et al., 1997).

Given the positive roles of sports activities in psychological regulation, emotion management, and social skills enhancement, there exists sufficient rationale to support the hypothesis that physical education, through engaging in sports activities, significantly predicts improvements in student mental health. Therefore, we propose Hypothesis H1: Physical education has a significant positive effect on the mental health of university students.

### 2.2 The mediating effect of social support

Social support refers to the external material and emotional assistance individuals receive through their social connections, providing a sense of security, belonging, and self-worth (Simpson et al., 2021). According to the Self-Determination Theory, social environments that fulfill basic psychological needs for autonomy, relatedness, and competence can enhance individual motivation and its transformation. Social support has a significantly positive predictive effect on the psychological health of college students; higher levels of social support are associated with better psychological well-being across various subdomains. College students can learn about psychological health from friends and classmates and develop cooperation, negotiation, and social skills through project-based or self-directed learning. Students with a high degree of self-focus can perceive more social support, thereby improving their psychological health and life satisfaction.

As the role of social support in the psychological health of college students gains attention, it becomes evident that physical education and social support are positively correlated. Higher levels of physical exercise among college students are associated with greater social support scores (Ünlü, 2023). Engagement in physical activities can enhance daily exercise behaviors, increase interaction frequencies among students, and strengthen social networks, thereby providing more social and emotional resources. The various forms of assistance and support college students receive in sports settings, including subjective emotional and psychological support, objective material support, and the effective utilization of these supports, impact the psychological benefits of exercise. Hypothesis H2 is proposed: Social Support has a positive mediating effect between physical education and the psychological health of university students.

### 2.3 Mediating effect of exercise behavior

Exercise behavior refers to physical activity undertaken during leisure time, characterized by specific duration, frequency, and intensity (Biddle and Nigg, 2000). Extensive research has demonstrated that engaging in sports activities effectively reduces stress, symptoms of depression, social anxiety, and feelings of loneliness (Xiang et al., 2020; Singh et al., 2023). A supportive sports environment can enhance the enthusiasm of middle school students for participating in sports, just as nurturing methods in university sports education can motivate elementary students’ participation.

The Self-Determination Theory and the Socio-Ecological Model elucidate the mediating role of exercise behavior between physical education and mental health. Self-Determination Theory posits that the satisfaction of the three basic psychological needs—autonomy, competence, and relatedness—can internalize extrinsic motivation into intrinsic motivation, enhancing the voluntary engagement in physical activities (Gagné et al., 2022; Ryan and Vansteenkiste, 2023). In the university sports environment, parents’ emotional and behavioral support can ignite and nurture students’ intrinsic motivation to participate in sports, fulfilling these basic psychological needs and, consequently, improving mental health. The Socio-Ecological Model comprehensively summarizes how multiple factors within the university environment influence students’ exercise behavior and mental health (Langille and Rodgers, 2010). Physical environments in universities can have both positive and negative effects on students’ exercise behavior and mental health. Excessive use of electronic devices can increase sedentary activity and lead to issues like internet addiction. Contrastingly, the university sports environment can mitigate the adverse effects of media exposure and foster positive influences on students’ exercise behaviors and mental health. Based on these observations, we propose Hypothesis H3: Exercise behavior mediates positively between physical education and student mental health.

### 2.4 Chain mediating effect of social support and exercise behavior

In the relationship between physical education and student mental health, social support and exercise behavior together constitute a chain mediation model. This model can be analyzed from the perspectives of psychosocial theory and behavioral development theory. Initially, physical education enhances students’ social support by organizing team activities and projects that help them develop cooperation and communication skills, build close social relationships, and strengthen their sense of belonging to a group. It also provides a relaxed environment for practicing social skills, fostering team cohesion through shared goals and mutual support. During sports activities, students motivate each other and receive emotional support from teachers and coaches, which plays a positive role in boosting self-esteem and confidence. Furthermore, extracurricular activities expand students’ social networks, thereby enhancing their overall social support. According to Behavioral Development Theory (Biddle and Nigg, 2000), favorable social environments and personal support increase the likelihood of maintaining healthy behaviors, such as regular exercise. Regular physical activity not only improves physical health but also enhances mental states through physiological mechanisms, like the release of endorphins, reducing depression and anxiety levels, and directly boosting mental health. Therefore, in this chain mediation model, enhanced social support strengthens exercise behavior, and more frequent exercise further improves students’ mental health. This process illustrates the positive impact of physical education on student mental health, where social support and exercise behavior play key mediating roles. Consequently, we propose Hypothesis H4: Social Support and exercise behavior act as a chain mediating between physical education and student mental health.

**Figure 1:**
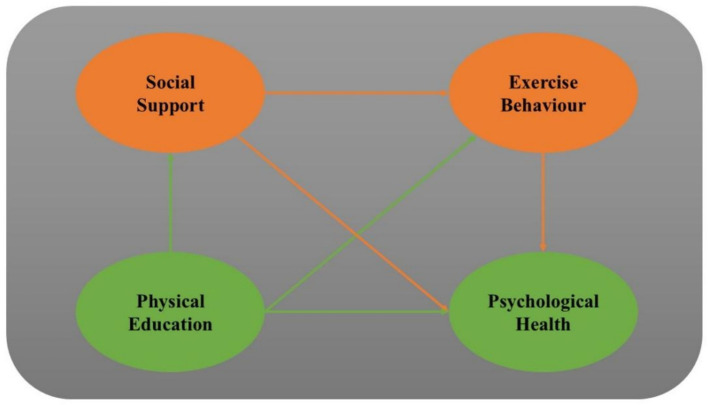
Mechanism diagram.

The mechanism diagram is shown in Figure 1.

## 3 Methods

### 3.1 Study participants

**Figure 2:**
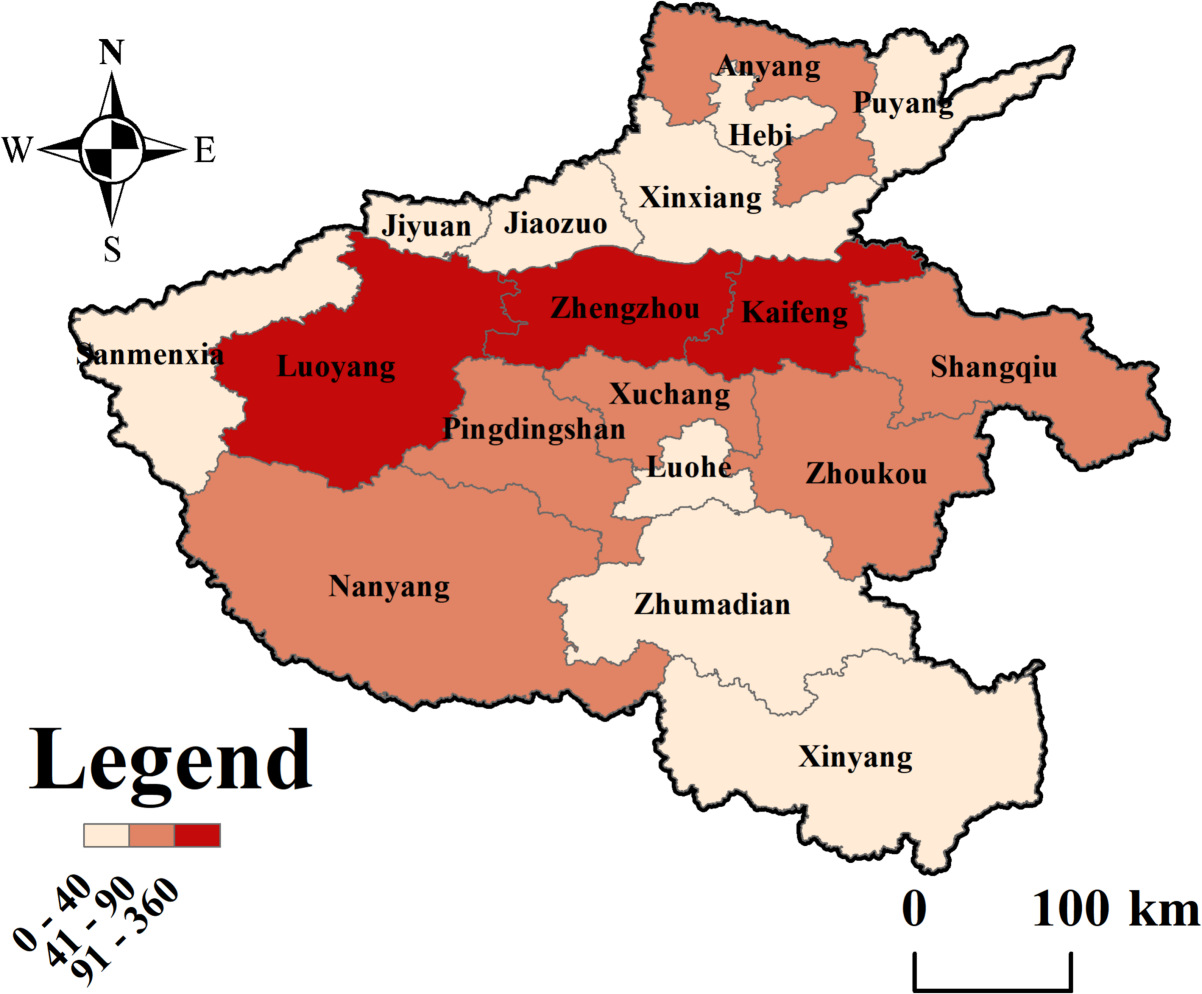
Regional distribution map.

To ensure the generalizability of the conclusions, a simple random sampling method was employed, and questionnaires were distributed to universities across Henan Province through a combination of online and offline methods. Due to the clustering of universities in certain regions, the survey mainly focused on Zhengzhou, Kaifeng, and Luoyang, where the number of universities is relatively high. Initially, 1,895 questionnaires were collected. The data were then screened, and questionnaires were excluded based on the following criteria: (1) missing responses for more than 30% of the items; (2) missing demographic information such as gender or age; (3) uniform responses across all items within the same scale. After this screening process, 1,437 valid questionnaires were retained, resulting in an effective response rate of 75.83%. Among the participants, 769 (53.51%) were male, and 668 (46.49%) were female. The demographic distribution of the survey participants is shown in Table 1. To make the survey questionnaire data more intuitive, ArcGIS 10.7 software was used for visualization, as shown in Figure 2. The study relies solely on publicly available data and does not involve human or animal participants, thus no ethical approval is required.

**Table 1:** Demographic distribution of survey participants.

Variable	Category	Number	Percentage (%)
Gender	Male	769	53.51
	Female	668	46.49
Grade	Freshman	327	22.76
	Sophomore	493	34.31
	Junior	281	19.55
	Senior	336	23.38
Origin	Urban	632	43.98
	Rural	805	56.02
Only child	Yes	790	54.98
	No	647	45.02
Total		1,437	100

### 3.2 Research methods

#### 3.2.1 Physical education questionnaire

To better assess the level of physical education, this study developed a Physical Education Satisfaction Scale based on the SERVQUAL model and inspired by research from Yuan et al. (2022) (Liu and Chung, 2014; Trigueros et al., 2019). The questionnaire comprises five sections, with a total of 24 items covering “Sports Facilities,” “Physical Education Teaching,” “Extracurricular Sports,” “Perceived Value,” and “Overall Satisfaction.” The test–retest reliability of the scale is 0.82 and the Cronbach’s *α* coefficient is 0.963. The model fit indices are χ^2^/df = 2.221, RMSEA = 0.043, CFI = 0.913, GFI = 0.921, TLI = 0.911, and IFI = 0.946, indicating a high model fit.

#### 3.2.2 Mental health diagnostic test

The Revised SCL-90 Scale (Derogatis and Unger, 2010) was utilized to assess the psychological health of university students, which measures nine dimensions: psychiatric, emotional, cognitive, consciousness, behavioral, lifestyle habits, interpersonal relationships, diet, and sleep. It uses a five-point Likert scale ranging from “none” to “severe.” In this study, “none” is scored as 1 with scores 2 to 5 indicating positive symptoms. The scale’s reliability is excellent (Cronbach’s *α* = 0.981), and model fit indices are satisfactory with *χ*^2^/df = 2.321, RMSEA = 0.045, CFI = 0.942, GFI = 0.931, TLI = 0.932, and IFI = 0.936.

#### 3.2.3 Social support level measurement

The measurement of social support drew from Anderson et al. (2024), encompassing 17 items across three dimensions: objective support, subjective support, and support utilization, scored on a five-point Likert scale (1–5). Higher scores represent higher levels of social support. The Cronbach’s *α* reliability coefficient is 0.952, and the model fit indices are χ^2^/df = 2.355, RMSEA = 0.047, CFI = 0.956, GFI = 0.928, TLI = 0.942, IFI = 0.919, indicating a high model fit.

#### 3.2.4 Exercise behavior level measurement

Using the International Physical Activity Questionnaire (Craig et al., 2003; Lee et al., 2011), student exercise behaviors were assessed across four items such as “How intense is your physical exercise?” The scale evaluates the quantity of exercise based on the intensity, duration, and frequency (Exercise Quantity = Intensity × Time × Frequency). Intensity and frequency are scored from 1 to 5, while time is scored from 0 to 4, with the maximum score being 100 points and the minimum 0. Exercise levels are categorized as low (≤19), moderate (20–42), and high (≥43). This scale’s Cronbach’s *α* reliability coefficient is 0.967, with model fit indices of χ^2^/df = 2.551, RMSEA = 0.046, CFI = 0.938, GFI = 0.927, TLI = 0.942, IFI = 0.963, demonstrating a high degree of model fit.

#### 3.2.5 Data processing

Data were analyzed using SPSS 26.0, employing Pearson correlation, regression, and mediation analyses. Mediation analysis was conducted using the Boot Strap method with the PROCESS (Version 4.1) plugin. For analyzing indirect effects, Model 6 was specified in PROCESS with the following parameters: X = physical education, M1 = social support, M2 = exercise behavior, Y = psychological health, with Boot Strap samples set to 5,000.

## 4 Results

### 4.1 Test for common method bias

Given the possible influence of the survey environment, instructions, and context, common method bias (CMB) may be a concern when gathering data through questionnaires. A Harman’s single-factor test was performed using SPSS 26.0, conducting an unrotated principal component analysis of the data related to physical education, psychological health, social support, and exercise behavior. The analysis identified 20 factors with eigenvalues greater than 1, with the first factor explaining 30.90% of the variance, which is below the threshold of 40%. This suggests that common method bias is not present in the data of this study.

### 4.2 Analysis of the current status of physical education, psychological health, social support, and exercise behavior

A one-sample T-test with a test value of the median was conducted (Table 2). The results indicate that the scores for physical education, psychological health, and social support were significantly higher than the median, suggesting a strong approval of physical education among students, and indicating that both psychological health and social support are above average. However, the scores for exercise behavior were significantly lower than the median, with 78.16% of respondents reporting low levels of exercise (mean = 2.261, SD = 0.637), 12.81% reporting moderate levels (mean = 3.433, SD = 0.191), and only 9.03% engaging in high levels of exercise (mean = 4.177, SD = 0.416). These statistics confirm the lack of physical exercise among contemporary university students, indicating that the majority of them lack sufficient exercise and exhibit suboptimal physical fitness.

**Table 2:** One-sample T-test for physical education, psychological health, social support, and exercise behavior.

Variable	Mean	SD	*T*-value
Physical education	3.598	0.556	32.017^***^
Psychological health	3.567	0.652	23.147^***^
Social support	3.791	0.677	31.773^***^
Exercise behavior	2.741	0.865	−6.335^***^

“***” indicates *p* < 0.01.

### 4.3 Correlation analysis among variables

Pearson’s correlation analysis was conducted to investigate the relationships between physical education, psychological health, social support, and exercise behavior. Results revealed significant positive correlations among all these variables (Table 3).

**Table 3:** Correlation analysis of physical education, psychological health, social support, and exercise behavior.

Variable	1	2	3	4
University Phys. Ed.	1			
Psychological health	0.538^***^	1		
Social support	0.372^***^	0.481^***^	1	
Exercise behavior	0.269^***^	0.339^***^	0.849^***^	1

“***” indicates *p* < 0.01.

Furthermore, regression analyses were performed with physical education, social spport, and exercise behavior as independent variables, and psychological health as the dependent variable using a forced entry method. The results, shown in Table 4, indicate significant impacts of physical exercise (*β* = 0.632), social support (*β* = 0.576), and psychological resilience (*β* = 0.631) on the emotional and social capabilities of young adults with each explaining 51.1, 44.1, and 43.8% of the variance, respectively.

**Table 4:** Separate regression analyses of physical education, social support, and exercise behavior on psychological health.

Variable	*β*	*SE*	*β*	*T*	*F*	*R^2^_adj_*
Constant	2.455	0.055				
University Phys. Ed.	0.378	0.023	0.632^***^	19.633	534.872	0.633
Constant	1.644	0.144				
Social support	0.463	0.038	0.576^***^	19.234	477.239	0.459
Constant	1.389	0.146				
Exercise behavior	0.623	0.047	0.631^***^	18.071	18.765	0.476

*R*^2^_adj_ refers to the adjusted *R*^2^; “***” indicates *p* < 0.01.

### 4.4 Testing the direct and indirect effects of physical education on student psychological health

Using physical education as the independent variable and psychological health as the dependent variable, and incorporating social support and exercise behavior as mediating variables, a series of regressions were conducted controlling for demographic variables like gender. This analysis was performed using SPSS 26.0 and the PROCESS 3.4 plugin to test the chain mediating effects between physical education and psychological health; results are presented in Table 5. The analysis proceeded in three steps:

1. Initializing the regression model: starting with psychological health as the dependent variable, demographic controls such as gender, class year, place of origin, and only child status were entered to account for their potential influence on youth psychological health.
2. Testing direct effects: physical education was introduced into the regression model to examine its total direct effect on psychological health after controlling for demographic variables. It was found to significantly and positively predict psychological sounds (*β* = 0.622, *p* < 0.001), confirming Hypothesis H1.
3. Testing mediating effects: social support and exercise behavior were sequentially added to the model to test their mediation between physical education and psychological health. Results indicated significant positive predictions for psychological health from both social adaptibility (*β* = 0.277, *p* < 0.001) and exercise behavior (*β* = 0.357, *p* < 0.001). It was also found that physical education can significantly predict social support (*β* = 0.523, *p* < 0.001) and exercise behavior (*β* = 0.473, *p* < 0.001), with social support significantly foretelling exercise behavior (*β* = 0.578, *p* < 0.001). These coefficients indicate significant chain mediating effects, verifying Hypotheses H2 and H3.

**Table 5:** Mediation regression results.

Variable	Psychological health		Social support		Exercise behavior		Psychological health	
	*β*	*t*	*β*	*t*	*β*	*t*	*β*	*t*
Gender	0.048	1.324	0.892	2.983	0.031	0.882	0.045	0.732
Grade	0.002	0.344	0.025	0.126	−0.038	−1.376	0.045	0.666
Origin	−0.071	−1.174	0.028	0.392	−0.028	0.532	−0.056	−1.012
Only child	--0.034	−0.468	0.048	1.356	0.003	0.021	−0.039	−1.210
Physical education	0.622^***^	17.84	0.523^***^	19.43	0.277^***^	7.886	0.311^***^	6.234
Social support					0.578^***^	16.388	0.473^***^	9.032
Exercise behavior							0.357^***^	7.893
*R^2^*	0.441		0.278		0.661		0.512	
*△R^2^*	0.403		0.298		0.601		0.588	
*F*	40.235^***^		21.345^***^		85.893^***^		76.982^***^	

“***” indicates *p* < 0.01.

Mediation effect model testing with AMOS 26.0: The fit of the model was good: χ^2^/df = 2.678, RMSEA = 0.056, CFI = 0.943, GFI = 0.927, TLI = 0.942, IFI = 0.917. A Bootstrap confidence interval analysis with 5,000 samples confirmed significant total, direct, and mediating effects of physical education on psychological health.

It becomes evident that physical education can predict psychological health, and both social Support and exercise behavior serve intermediate mediating roles across three pathways. The total indirect effects were substantial (Effect size = 0.306, Bootstrap 95% CI not including 0: [0.312, 0.433], representing 55.84% of the total effect). Specifically:

Path 1: from physical education to social support to psychological health had an indirect effect of0.164, accounting for 29.93% of the total effect.
Path 2: from physical education to exercise behavior to psychological health had an indirect effect of 0.069, representing 12.59%.
Path 3: the chain mediation from physical education to social support to exercise behavior to psychological health had an indirect effect of 0.073, accounting for 13.32%.

**Figure 3:**
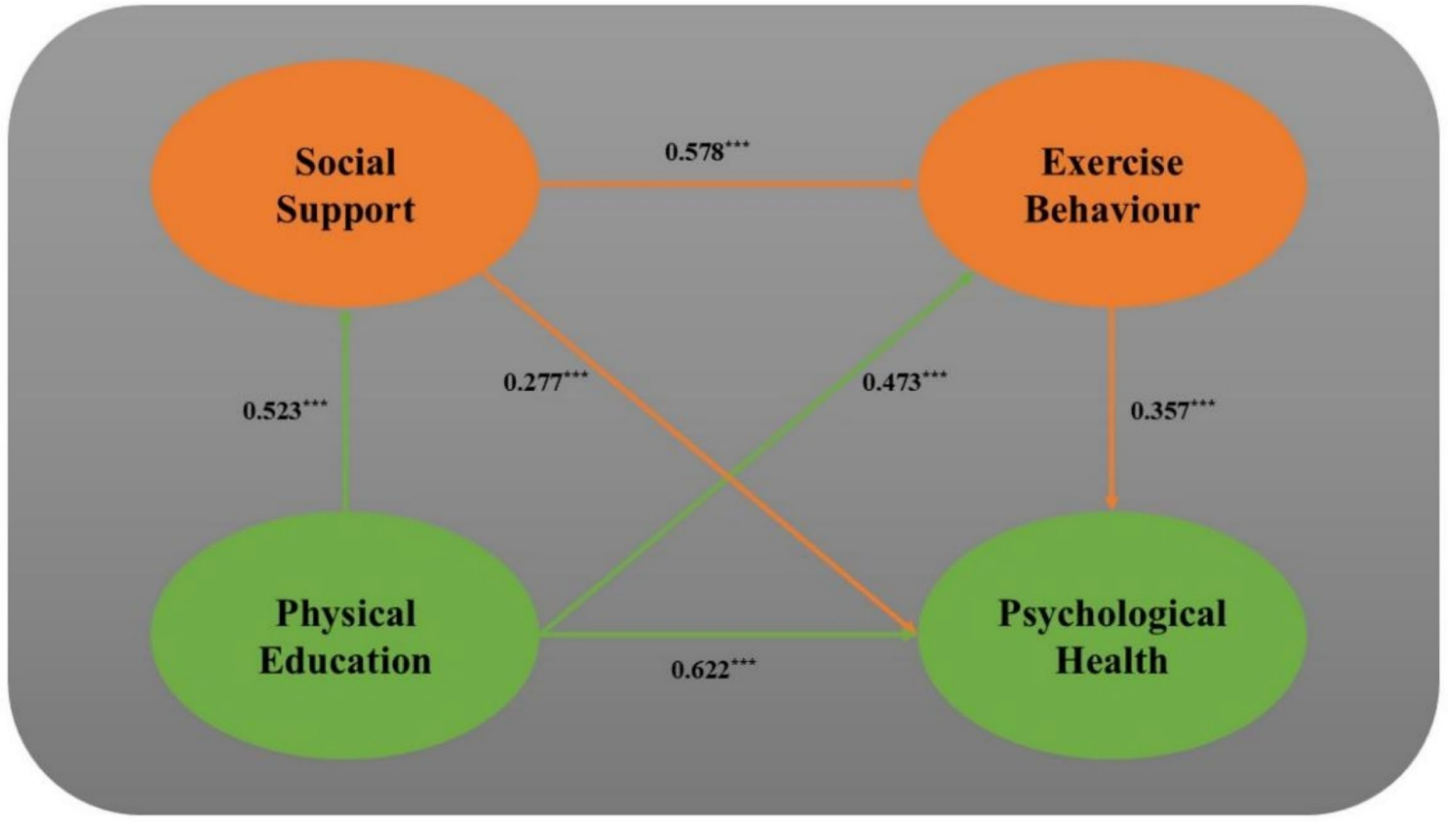
Path diagram of the impact of physical education on student psychological health.

These findings substantiate Hypothesis H4, illustrating the comprehensive impact of physical education on psychological health through social support and exercise behaviors. The chain mediation model is depicted in Figure 3. Formal reporting of these analyses underscores the critical linkages between physical education and key psychological outcomes in university settings (Table 6).

**Table 6:** Mediation effects of social support and exercise behavior.

Type	Path	Effect size	Standard error	LLCL	ULCL	Effect percentage (%)
Total		0.548	0.031	0.477	0.532	100
Direct	Direct Path	0.242	0.042	0.135	0.296	44.16
Total indirect		0.306	0.033	0.312	0.433	55.84
Indirect	Path 1	0.164	0.030	0.027	0.174	29.93
	Path 2	0.069	0.019	0.056	0.123	12.59
	Path 3	0.073	0.021	0.037	0.188	13.32

## 5 Discussion

This study elucidates the direct and indirect pathways through which physical education influences the psychological health of college students, representing a proactive exploration into the prevention and promotion of mental health development among this population. Theoretically, our findings enrich the understanding of the factors and mechanisms impacting psychological health and deepen the scholarly output on physical education. Practically, they underscore the importance of physical education programs in universities and offer new insights into enhancing student social support, fostering exercise habits, and preventing and mitigating mental health issues among students.

### 5.1 Direct effects of physical education on student psychological health

The findings indicate that physical education has a significant positive impact on the psychological health of students, with an effect size of 0.622, validating Hypothesis H1. The direct effects of physical education accounted for as much as 44.16% of the influence on psychological health, highlighting its pivotal role in the development of student mental wellness. Physical education, serving as a significant medium for students to engage in sports, is an effective way to foster physical and mental health. As primarily an activity-based educational initiative, it directly influences physiological bases and, owing to its integrative nature of physical and emotional interaction, it also possesses the capability to enhance psychological and moral qualities. The direct impact of physical education on psychological health can be explored through two dimensions: (1) Addressing psychological needs: frequently overlooked psychological needs of college students are a primary reason for poor mental health conditions. Regular physical exercises integrated into university education can promote skeletal development, enhance flexibility and endurance, boost immunity, decrease the risk of non-communicable diseases (such as obesity, hypertension, diabetes), improve sleep quality, and alleviate academic pressure and anxiety. This, in turn, helps students better manage challenges in their academic and personal lives. Importantly, physical education not only helps in shaping a healthy physique but also positively influences the satisfaction of psychological needs and the cultivation of self-confidence among students (Hu and Tang, 2022). (2) Emotional regulation and stress relief: physical education significantly affects emotional regulation and the release of psychological stress among college students. This mechanism of emotional adjustment works through promoting the secretion of neurotransmitters such as dopamine and serotonin, which help to alleviate negative emotions such as depression and anxiety. In addition, physical education plays a significant role in helping students develop focus, transform negative emotions, and enhance positive emotional experiences. From a more positive perspective, during competitive sports activities, students gradually learn how to cope with failure and face setbacks, viewing these challenges as opportunities for growth. This experience not only aids in cultivating an optimistic and positive attitude toward life but also teaches them to bravely confront and overcome various difficulties in life. Through these experiences, students’ resilience and ability to cope with adversity are effectively enhanced, thereby improving their overall psychological health. However, while physical education is generally beneficial to students’ mental health, it may also bring certain negative effects, such as excessive competition pressure, potential sports injuries, unhealthy social comparisons, fear of failure, and time management pressures (Wang et al., 2023). Additionally, negative feedback from teachers or peers might adversely affect students’ mental well-being. These factors could lead to anxiety, low self-esteem, and emotional exhaustion. Therefore, it’s essential to provide appropriate support and guidance when implementing physical education to mitigate these negative effects and ensure that students can benefit fully.

### 5.2 The mediating role of social support

Our findings indicate that physical education impacts student psychological health through social support, an indirect pathway, thereby confirming Hypothesis H2. In the relationship between physical education and mental health, social support plays a crucial mediating role. Through teamwork and collective training, physical education fosters social interaction among students, creating an environment rich in social support (Kiliç, 2021). Social support, which includes emotional support, informational support, and practical assistance, directly enhances mental health. Sports activities help students build deep emotional connections, provide psychological security, and reduce the risk of anxiety and depression. Meanwhile, the informational support and social skills gained through sports activities help improve psychological adaptability, bolster self-esteem, and increase self-efficacy, thereby indirectly promoting mental health. Additionally, practical support aids students in better handling life’s challenges and enhances life satisfaction.

However, social support can also have negative effects. Over-reliance on social support may lead to a lack of independence and diminished problem-solving abilities. Moreover, negative influences within social networks, such as peer pressure or the imitation of undesirable behaviors, could negatively impact mental health (Thio and Elliott, 2005). Furthermore, inappropriate social support or its absence at critical moments can result in greater emotional loss and psychological distress. Therefore, understanding and managing the dual effects of social support is essential for optimizing physical education and promoting mental health.

### 5.3 The mediating role of physical exercise

This study found that physical education indirectly influences mental health by promoting exercise behaviors, thus confirming hypothesis H3. Tian (2024) pointed out that exercise behavior plays a mediating role in mental health, providing support for our Hypothesis 3. Through physiological mechanisms, exercise behavior promotes the secretion of dopamine and serotonin, improving emotional state, alleviating stress and anxiety, and enhancing mental health. Furthermore, exercise behavior enhances students’ self-esteem and self-confidence by achieving personal goals and boosting self-efficacy, which contributes positively to mental health. Participation in individual or group sports also facilitates social interaction and the building of interpersonal networks, providing a foundation for emotional support and psychological adaptation, and strengthening students’ resilience to life stressors.

However, exercise behavior may also have negative effects. Excessive exercise can lead to physical injuries and increased psychological stress, which may harm health. Additionally, over-reliance on exercise behavior can result in an unbalanced lifestyle and may lead to feelings of isolation or unhealthy competition in social contexts. In physical education, it is essential to design exercise plans rationally to minimize these potential negative factors.

In summary, exercise behavior, through multiple effects, not only promotes students’ physical fitness but also significantly enhances mental health. In the realm of physical education, appropriate exercise behavior design and implementation are crucial to achieving physical health goals while avoiding unnecessary negative impacts, thereby fully supporting students’ mental health.

### 5.4 The chain mediation effect of social support and exercise behavior

The study revealed that, in addition to their individual mediating effects, social support and exercise behavior can jointly mediate the relationship between physical education and mental health, thus confirming hypothesis H4. In the realm of physical education, social support serves as a pivotal element. Through engaging in team sports and collaborative activities, students gain emotional and informational support from coaches and peers. This support not only bolsters students’ psychological security and sense of belonging but also lays a strong foundation for their enthusiastic participation in exercise activities.

Social support plays a crucial role in promoting exercise behavior by providing individuals with emotional and practical resources, enhancing their motivation and capacity to engage in physical activity. From a theoretical perspective, Social Cognitive Theory suggests that social influences, such as encouragement from peers, family, and community members, can boost self-efficacy and reinforce positive behaviors, including regular exercise (Wu et al., 2023). Empirical studies have shown that individuals who receive consistent support from their social networks—such as exercise reminders, companionship during activities, or recognition of achievements—are more likely to develop and maintain exercise habits.

As a direct beneficiary of social support, exercise behavior has a significant positive impact on mental health. Regular physical activity not only enhances students’ physical well-being but also stimulates the release of neurotransmitters like dopamine and serotonin, aiding in emotional regulation and stress management. Through continued participation in exercise, students gradually build self-efficacy and self-esteem, thus increasing their confidence—a critical psychological resource for sustaining mental health.

Nonetheless, social support and exercise behavior can sometimes have adverse effects. Excessive reliance on social support might lead to diminished independence and autonomy in students, weakening their problem-solving skills. Furthermore, students may face peer pressure or get drawn into unhealthy competition within social circles, placing a strain on their mental health. Overindulgence in exercise behavior can also result in physical injuries, heightened psychological stress, and negative health impacts.

From these observations, we can extract several crucial insights: First, physical education programs should aim to balance the social environment by fostering a robust social support network through well-structured team activities and interaction opportunities. Second, students should be encouraged to engage in various forms of exercise in moderation, thereby enhancing social interaction while simultaneously improving physical fitness. Lastly, policymakers in education should devise more nuanced interventions that address these potential challenges to comprehensively bolster students’ mental health. Adopting this holistic strategy not only guarantees well-rounded development but also equips students to better navigate future challenges.

### 5.5 Practical significance

The study reveals the pathways through which social support and exercise behavior influence the relationship between physical education and college students’ mental health, offering valuable insights for sports educators, psychology professionals, and policymakers. For sports educators, particularly teachers, integrating social practice into the curriculum and collaborating with communities, families, or other organizations can create positive emotional experiences for students, effectively reducing mental stress and enhancing psychological health. For psychology professionals, the study confirms the critical role of social support and exercise behavior in improving mental health, broadening treatment options, and encouraging deeper consideration of social factors in patient care, thereby promoting diversified therapeutic approaches. For policymakers, the findings provide actionable insights that can inform public health strategies and policy development.

### 5.6 Limitations

1. Sample size and diversity: the sample size in this study is relatively small and primarily focused on students from Henan Province. Future research should expand the sample size and diversity to include students from various regions to comprehensively explore the universal impact of physical exercise on college students’ psychological health.
2. Institutional variations: the study mainly includes students from public institutions. Given that educational groups and characteristics may vary by type of institution, future research should consider focusing on specific types of educational institutions.
3. Research design: this study primarily uses a cross-sectional design, making it difficult to establish causal relationships between variables. Future research should combine longitudinal studies and experipsychological designs to explore causal relationships among variables more effectively.

## 6 Conclusion and recommendations

### 6.1 Conclusion

This study, from the perspective of physical education in higher education institutions, explores a potential solution to improve college students’ mental health. It also provides empirical support for enhancing students’ social support and fostering positive exercise behaviors. The following conclusions were drawn:

1. There is a significant correlation between physical education in higher education, social support, exercise behavior, and college students’ mental health.
2. Physical education in higher education can significantly and positively promote college students’ mental health and is a crucial intervention variable influencing their mental health development.
3. Social support and exercise behavior not only serve as simple mediators in the impact of physical education on college students’ mental health but also influence mental health through a chain effect involving social support and exercise behavior. This indirect mediation effect plays a significant role in the development of college students’ mental health.

### 6.2 Recommendations

Based on the study’s conclusions, the following recommendations are proposed:

1. Integrate teaching curricula: combine physical education and mental health education to promote holistic student development. Comprehensive courses should cover both physical and mental health, fostering a supportive learning environment. Tailor curricula to students’ needs and innovate teaching methods to ensure effective outcomes.
2. School-community collaboration: schools and communities should work together to enhance students’ mental health. Schools can organize activities such as sports meets and open days, involving parents and friends. Communities can provide sports training and guidance, helping students develop exercise habits and allowing them to feel the warmth and care of the community.
3. Physical education teacher training: schools should provide training for physical education teachers on mental health topics, including identifying and addressing psychological issues, emotion management, and stress coping. This helps teachers integrate mental health education into physical education, observe students’ emotional changes, and apply effective interventions. Regular workshops and expert guidance can further enhance teachers’ skills.

## Data Availability

The raw data supporting the conclusions of this article will be made available by the authors, without undue reservation.
